# Comparison of Nasal and Oropharyngeal Bleeding in Video Laryngoscopy Versus Direct Laryngoscopy for Nasotracheal Intubation in Maxillofacial Trauma: A Randomized Controlled Trial

**DOI:** 10.1155/anrp/7797828

**Published:** 2025-07-03

**Authors:** Ayman Mohamady Eldemrdash, Mohamed A. Alazhary, Zaher Zaki Zaher, Tarek S. Hemaida, Mohammed Essam Yahia, Soudy S. Hammad

**Affiliations:** Department of Anesthesia and Intensive Care Unit, Faculty of Medicine, Aswan University, Aswan, Egypt

**Keywords:** airway management, bleeding severity, direct laryngoscopy, maxillofacial trauma, nasotracheal intubation, video laryngoscopy

## Abstract

**Background:** Nasotracheal intubation (NTI) is commonly used in maxillofacial trauma but carries a high risk of bleeding and airway complications, particularly with direct laryngoscopy (DL). Video laryngoscopy (VL) provides better glottic visualization and may reduce airway trauma. This study compares VL and DL for NTI in maxillofacial trauma patients, focusing on bleeding severity, intubation efficiency, and complications.

**Methods:** This randomized controlled trial included 64 patients undergoing NTI for maxillofacial trauma, randomly assigned to VL or DL. The primary outcome was nasal and oropharyngeal bleeding severity, assessed using Fromme's scale. Secondary outcomes included first-pass success rate, intubation time, need for adjunctive maneuvers (Magill forceps and cervical spine extension), and intubation-related complications. All intubations were performed under general anesthesia following standardized airway preparation.

**Results:** VL resulted in significantly lower nasal and oropharyngeal bleeding severity, with no bleeding (score 0) observed in 43.8% of the VL patients versus 12.5% of the DL group (*p*=0.005). VL also resulted in shorter intubation times (51.9 ± 7.9 s vs. 58.1 ± 8.7 s; *p*=0.003). The need for adjunctive maneuvers was significantly lower in the VL group (*p* < 0.001), and severe complications such as fractured teeth or deep lip injuries occurred more frequently in the DL group (*p*=0.02). The first-pass success rate was higher in the VL group (96.9%) than in the DL group (78.1%) though the difference was not statistically significant (*p*=0.058).

**Conclusion:** VL demonstrated superior intubation efficiency and reduced bleeding severity compared with DL in maxillofacial trauma patients. Given its safety advantages and reduced need for adjuncts, VL appears to be a preferable technique for NTI in maxillofacial trauma though further multicenter studies are ensured.

**Trial Registration:** ClinicalTrials.gov identifier: NCT06386757

## 1. Introduction

Maxillofacial trauma presents significant challenges in airway management due to anatomical distortion, bleeding risk, and associated cervical spine injuries. In such cases, nasotracheal intubation (NTI) is often preferred over orotracheal intubation as it provides better surgical access, particularly in mandibular fractures and occlusal alignment procedures [1]. However, NTI is associated with a high risk of nasal and oropharyngeal bleeding, which may obscure visualization, prolong intubation attempts, and increase complications such as aspiration and airway obstruction [2].

Traditionally, direct laryngoscopy (DL) has been the standard approach for NTI. However, it requires a direct line of sight to the glottis, which can be problematic in trauma patients with swelling, limited neck mobility, or ongoing bleeding [3]. In addition, the frequent need for adjunctive maneuvers, such as the use of Magill forceps to guide the endotracheal tube (ETT), increases the risk of mucosal trauma and epistaxis [4].

In contrast, video laryngoscopy (VL) has emerged as a promising alternative, offering an indirect and magnified view of the glottis without requiring significant neck extension [5]. Studies have shown that VL improves first-pass success rates, minimizes airway trauma, and reduces the need for additional airway manipulation [6]. Moreover, recent meta-analyses suggest that VL reduces the incidence and severity of bleeding during NTI, making it particularly beneficial in maxillofacial trauma patients [7]. However, despite these advantages, there remains a need for direct comparative studies between VL and DL in trauma patients undergoing NTI to establish evidence-based guidelines for best practice [8].

This study aims to compare VL and DL for NTI in maxillofacial trauma patients, with the primary objective of assessing nasal and oropharyngeal bleeding severity using Fromme's scale [9]. Secondary objectives include evaluating first-pass success rates, intubation time, the need for adjunctive maneuvers (Magill forceps, cervical spine extension), and intubation-related complications. This research seeks to provide a clearer understanding of the optimal technique for NTI in maxillofacial trauma and contribute to improved patient outcomes.

## 2. Methods

### 2.1. Study Design and Setting

This prospective, randomized clinical trial was conducted at Aswan University Hospital from April 2024 to February 2025. The study received ethical approval from the Institutional Review Board (IEC Ref no: Asw. Uni./911/3/24). Written informed consent was obtained from all participants prior to enrollment.

### 2.2. Participants

#### 2.2.1. Inclusion Criteria

Adult patients aged (18–70 years) undergoing NTI for maxillofacial trauma surgery, American Society of Anesthesiologists (ASA) physical status I or II, and body mass index (BMI) < 35 kg/m^2^.

#### 2.2.2. Exclusion Criteria

Bleeding disorders or current use of anticoagulants/antiplatelets; severe nasal septal deviation, nasal polyps, or anatomical airway obstruction; predicted difficult airway requiring fiberoptic intubation; ASA physical status III or IV; and emergency cases, defined as those requiring intubation within 6 h of hospital admission or performed without standard preoperative evaluation and airway preparation, were excluded to ensure procedural consistency and reduce clinical variability.

### 2.3. Randomization and Blinding

Patients were randomly assigned in a 1:1 ratio to either the VL group or DL group using a computer-generated randomization sequence. Allocation concealment was ensured using sealed, opaque envelopes.

Due to the nature of the procedure, the performing anesthetist was not blinded. However, outcome assessors evaluating bleeding severity and intubation-related complication were blinded to the technique used to minimize bias.

### 2.4. Preoperative Airway Preparation

All patients underwent standard airway assessment, including nasal patency evaluation, interincisor distance measurement, Mallampati classification, and history of prior nasal trauma. To reduce mucosal congestion selected nostril was pre-treated with oxymetazoline spray (0.05%). In addition, 2% lidocaine gel was applied to lubricate the ETT.

### 2.5. Anesthesia and Intubation Procedure

General anesthesia was induced using fentanyl (2 μg/kg), propofol (2.5 mg/kg), and rocuronium (0.6 mg/kg) for muscle relaxation. Patients underwent preoxygenation with 100% oxygen for 3 minutes before intubation, followed by mechanical ventilation.

For NTI, a standardized protocol was employed. A well-lubricated gum-elastic bougie was introduced through the nasal passage into the pharynx. The glottis was visualized using either a C-MAC video laryngoscope (VL group) or a Macintosh direct laryngoscope (DL group), guiding the bougie into the trachea. Magill forceps were used if necessary. The ETT was then advanced over the bougie into the trachea, after which the bougie was removed. Confirmation of correct tube placement was done.

  VL group: intubation was performed using a C-MAC video laryngoscope with a gum-elastic bougie and Magill forceps as needed to guide the tube.
  DL group: intubation was performed using a Macintosh direct laryngoscope with a gum-elastic bougie and Magill forceps as needed to guide the tube.

If intubation required more than three attempts or oxygen saturation dropped below 90%, the procedure was abandoned in favor of the ASA difficult airway algorithm.

The severity of nasal and oropharyngeal bleeding was assessed using the Fromme's ordinal bleeding scale (0–5), a tool originally validated for grading surgical field bleeding during endoscopic sinus procedures [9]. Although not specifically developed for airway assessments, the scale's structure based on suction frequency and its impact on visual clarity makes it well suited for evaluating bleeding during NTI, where glottic visualization is critical. In this study, the scale was applied without modification due to its practicality, ease of use, and reproducibility. It also enabled consistent grading by blinded assessors. Notably, similar ordinal bleeding scales have been utilized in prior airway research to overcome the lack of a universally accepted tool for quantifying bleeding during intubation (Wang et al. and Liu et al. [7, 8]).

### 2.6. Outcome Measures

#### 2.6.1. Primary Outcome

Severity of nasal and oropharyngeal bleeding was assessed using Fromme's ordinal bleeding scale (0–5).

◦  0: no bleeding.
◦ 1: minimal bleeding, no suction required.
◦ 2: mild bleeding, occasional suction required.
◦ 3: moderate bleeding, frequent suction required.
◦ 4: severe bleeding, compromising intubation.
◦ 5: massive bleeding, making intubation impossible.

#### 2.6.2. Secondary Outcomes

1. First-pass success rate: successful intubation on the initial attempt, with a second attempt defined as starting after correction of desaturation.
2. Intubation time: duration from bougie insertion to ETT cuff inflation.
3. Adjunctive maneuvers: use of Magill forceps or cervical spine extension when indicated.
4. Intubation-related complications: categorized as severe (e.g., fractured teeth and deep lip injuries) or mild (e.g., mucosal trauma, bruises, and transient desaturation < 90%) based on clinical relevance and potential for long-term morbidity.

### 2.7. Sample Size

Sample size calculation was performed using G∗Power (Version 3.1.3). Based on a pilot study, a mean difference of 0.7 in bleeding severity scores with a pooled standard deviation of 0.9 required 29 patients per group to achieve 80% power (β = 0.2 and α = 0.05). Allowing for a 10% dropout rate, 32 patients per group (total 64 patients) were included. Data were analyzed using *R* (Version 4.4.2). Independent *t*-tests were used for continuous variables. Chi-square tests or Fisher's exact tests were used for categorical variables. A *p* value < 0.05 was considered statistically significant.

### 2.8. Statistical Analysis

Continuous variables (e.g., intubation time) were analyzed using independent *t*-tests (normally distributed data) or Mann–Whitney *U* tests (non-normally distributed data). Categorical variables (e.g., first-pass success rate and bleeding severity categories) were analyzed using Chi-square tests or Fisher's exact tests (if expected cell counts were < 5). Effect sizes were calculated using Cohen's d for continuous variables and odds ratios (ORs) with 95% confidence intervals (CIs) for categorical comparisons. Missing data were handled using multiple imputation if > 5% of values were missing; otherwise, a complete case analysis was performed. Subgroup analyses were conducted based on the fracture type (mandibular, zygomatic, maxillary, and pan facial) and use of Magill forceps (required vs. not required). A *p* value < 0.05 was considered statistically significant.

## 3. Results

**Figure 1:**
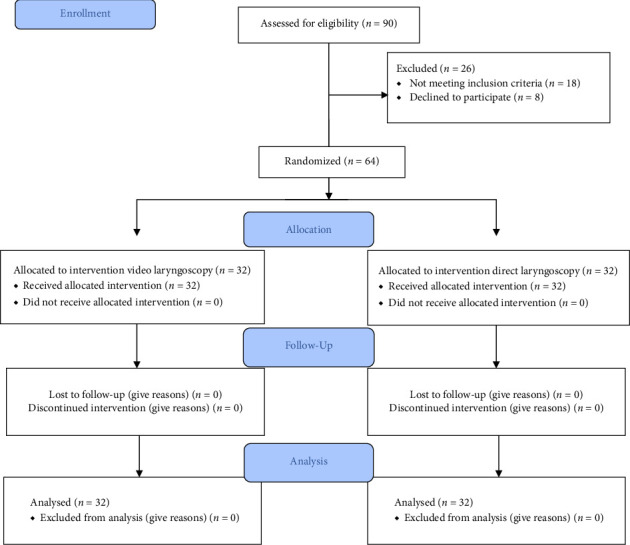
CONSORT flowchart.

A total of 90 patients were assessed for eligibility, with 26 excluded due to not meeting inclusion criteria (*n* = 18) or declining participation (*n* = 8). The remaining 64 patients were randomly assigned equally to the VL group (*n* = 32) and DL group (*n* = 32). All patients completed the study without protocol deviations (Figure 1).

Baseline demographic and clinical characteristics were comparable between the two groups, with no significant differences in age, BMI, ASA classification, smoking status, or fracture type (*p* > 0.05) (Table 1).

The overall incidence of nasal and oropharyngeal bleeding was significantly lower in the VL group (56.2%) compared with that of the DL group (87.5%) (*p*=0.006). Among patients who experienced bleeding, the severity of nasal and oropharyngeal bleeding, assessed using Fromme's ordinal bleeding scale, was significantly lower in the VL group compared with that of the DL group (*p*=0.005). No bleeding (score 0) was observed in 43.8% of the VL patients versus 12.5% of the DL patients. Minimal to mild bleeding (scores 1-2) occurred in 56.2% of the VL patients versus 84.4% of the DL patients. Moderate bleeding (score 3) was noted in 3.1% of the DL patients but not in VL patients. No cases of severe or massive bleeding (scores 4-5) were reported in either group (Table 2).

**Figure 2:**
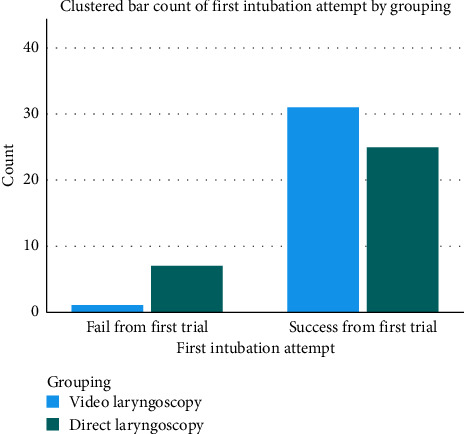
Fail and success from first-trial intubation.

Only one patient in the VL group required a second attempt (3.1%), compared with seven patients in the DL group (21.9%). The first-pass success rate was higher in the VL group (96.9%) compared with the DL group (78.1%); however, this difference did not reach statistical significance (*p*=0.058). All patients were successfully intubated within three attempts (Figure 2).

**Figure 3:**
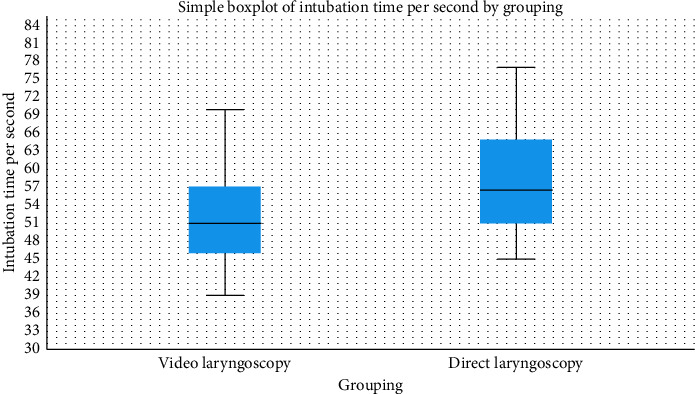
Intubation time per second by grouping.

Mean intubation time was significantly shorter in the VL group (51.9 ± 7.9 s) compared with the DL group (58.1 ± 8.7 s) (*p*=0.003). Cohen's effect size (*d* = −0.75 [95% CI: −1.26, −0.24]) suggests a clinically meaningful reduction in intubation time with VL (Figure 3).

The use of Magill forceps and cervical spine extension was significantly higher in the DL group (*p* < 0.001). Magill forceps were used in 46.9% of the VL patients versus 90.6% of the DL patients (*p*=0.0004). Cervical spine extension was required in 28.1% of the VL patients versus 81.2% of the DL patients (*p*=0.0005) (Table 3).

Severe intubation-related complications were more frequent in the DL group (43.7%) than in the VL group (15.6%) (*p*=0.02). Fractured teeth and deep lip lacerations were the most commonly observed severe complications. Few cases of mild complications were recorded in either group, including superficial mucosal injury, bruising, and transient desaturation (SpO_2_ < 90%) that resolved within 2 min without intervention.

## 4. Discussion

The findings of this study support the growing body of evidence that VL is superior to DL for NTI in maxillofacial trauma patients, particularly in reducing bleeding severity, improving intubation efficiency, and minimizing complications, making it a safer and more effective alternative.

One of the most clinically relevant finding of this study was the significantly lower incidence of nasal and oropharyngeal bleeding in the VL group compared with the DL group. Nearly half of VL patients (43.8%) experienced no bleeding whereas this was observed in only 12.5% of DL patients. This aligns with recent trials, indicating that VL minimizes mucosal trauma by providing better visualization and reducing forceful maneuvers during intubation [2]. Consistent with our findings, a meta-analysis by Zhang et al. [10] found that VL reduced epistaxis rates by 40% compared to DL, with significantly fewer cases of moderate-to-severe bleeding. Similarly, a 2023 randomized trial found that reported minimal epistaxis in video-assisted intubation, with no cases of severe bleeding [7]. However, a study by Lee et al. [11] reported no significant difference in bleeding severity between VL and DL in a mixed cohort of trauma and nontrauma patients. This discrepancy may be attributed to differences in patient selection, intubation techniques, or operator experience, highlighting the need for further investigation. This reduction in bleeding is likely attributed to decreased reliance on Magill forceps, which have been shown to contribute to mucosal trauma and hemorrhage [6]. By minimizing forceful manipulations, VL reduces the risk of excessive bleeding, airway obstruction and prolonged intubation attempts.

This study demonstrated that VL improved procedural efficiency, as evidenced by the significantly shorter intubation time in the VL group (51.9 ± 7.9 s vs. 58.1 ± 8.7 s). This difference is both statistically and clinically significant, as prolonged intubation can increase the risk of desaturation, aspiration, and hemodynamic instability. Our findings are consistent with previous research published in CHEST reported that the mean duration of laryngoscopy was 48 s in the VL group and 98 s in the DL group, resulting in a mean difference of 50 s [12] and also by Park et al. [13], which demonstrated that VL reduces external airway manipulations by 55%, thereby decreasing patient discomfort, intubation time, and trauma-related complications. Although the first-pass success rate was higher in the VL group (96.9%) compared with the DL group (78.1%), this difference did not reach statistical significance (*p*=0.058) but the trend suggests a clinically meaningful advantage, as multiple intubation attempts are associated with increased airway trauma, aspiration risk, and prolonged hypoxia. While this trend suggests a potential advantage of VL, it does not provide conclusive evidence of superiority, and further studies with larger sample sizes are needed to confirm this finding. However, Smith et al. [14] found no significant difference in first-pass success rates between VL and DL in trauma patients, possibly due to variability in airway management protocols or the use of different VL devices. These mixed findings indicate that while VL has potential advantages, its effectiveness may depend on patient-specific and operator-dependent factors.

A significant advantage of VL in this study was the reduced need for adjunctive maneuvers, including Magill forceps use and cervical spine extension. In the VL group, Magill forceps were required in only 46.9% of the patients, compared with 90.6% in the DL group (*p*=0.0004). Cervical spine extension was needed in 28.1% of the VL patients versus 81.2% in DL patients (*p*=0.0005). This is an important finding because reducing these maneuvers minimizes the risk of additional airway trauma and cervical spine instability, particularly in trauma patients with suspected cervical spine injuries. Nguyen et al. demonstrated that eliminating Magill forceps significantly reduced intubation time and the risk of mucosal injury, supporting our results [15].

The incidence of severe intubation-related complications was significantly lower in the VL group (15.6%) compared with the DL group (43.7%) (*p*=0.02). The most common complications in the DL group were fractured teeth, deep lip injuries, mucosal trauma, and bruising, reinforcing VL's safety advantage. These findings are supported by a systematic review by Liu et al. [8], which found that VL significantly reduces airway trauma, soft tissue injuries, and dental fractures compared with DL. Similarly, a prospective study by Brown et al. reported a 35% reduction in airway injuries with VL, further validating our results [6]. Notably, no cases of desaturation (SpO_2_ < 90%) were recorded in either group, suggesting that both techniques maintained adequate oxygenation throughout the procedure. However, the higher number of multiple intubations attempts in the DL group suggests a greater potential risk for desaturation in real-world settings.

Given the clear advantages of VL—reduced bleeding, improved success rates, shorter intubation times, and fewer complications—we recommend its routine adoption for NTI in maxillofacial trauma patients. However, further multicenter randomized controlled trials (RCTs) are needed to validate these findings and optimize best-practice guidelines.

Although this study provides strong evidence supporting VL for NTI in maxillofacial trauma, several limitations must be considered. First, it was conducted at a single institution, which may limit generalizability, highlighting the need for multicenter RCTs to validate these findings across diverse patient populations. Second, the study did not account for operator experience differences between VL and DL, which may influence intubation success and complication rates; future research should stratify outcomes based on anesthetist expertise. In addition, the study focused solely on immediate procedural outcomes, leaving postoperative complications such as airway edema, nasal mucosal healing, and patient-reported discomfort unexamined. Long-term follow-up studies are needed to assess these outcomes comprehensively. Finally, the economic feasibility of VL was not evaluated despite its higher cost compared with DL. To ensure broader adoption, cost-effectiveness studies should determine whether VL provides sufficient clinical benefits to justify its expense, particularly in resource-constrained settings [16].

Conclusion, VL is superior to DL for NTI in maxillofacial trauma, offering less bleeding, faster intubation, reduced adjunctive maneuver use, and fewer complications, making it the preferred technique in trauma settings where airway bleeding and cervical spine stability are concerns. Given these clear clinical advantages, VL should be the standard approach in trauma and surgical settings where NTI is required.

## Figures and Tables

**Table 1:** Comparison between studied groups regarding patient data.

	Video laryngoscopy *n* = 32	Direct laryngoscopy *n* = 32	*p* value
Age (years)	31 (11.4)	28.7 (8.8)	0.36
BMI	24.5 (3.4)	23.1 (2.8)	0.08
Gender (male)	26 (81.2%)	29 (90.6%)	0.4
Smoking	23 (71.9%)	25 (78.1%)	0.7
ASA I	23 (71.8%)	28 (87.5%)	0.47
Type of fracture			0.4
Zygoma	13 (40.6%)	11 (34.4%)	
Mandibular	14 (43.8%)	11 (34.4%)	
Maxilla	1 (3.1%)	4 (12.5%)	
Pan facial	4 (12.5%)	6 (18.8%)	

*Note:p* value was determined by one-way *t*-test to compare the difference in mean and chi square to compare the differences in frequencies between groups. Statistically nonsignificant: *p* value > 0.05.

**Table 2:** Comparison between studied groups regarding severity of bleeding.

	Video laryngoscopy *n* = 32	Direct laryngoscopy *n* = 32	*p* value
No bleeding	14 (43.8%)	4 (12.5%)	0.005
Minimal bleeding	16 (50%)	16 (50%)	
Mild	2 (6.2%)	11 (34.4%)	
Moderate	0 (0%)	1 (3.1%)	
Severe	0 (0%)	0 (0%)	
Massive	0 (0%)	0 (0%)	

*Note:p* value was determined by chi square to compare the differences in frequencies between groups. Statistically significant: *p* value< 0.05.

**Table 3:** Comparison between studied groups regarding first-attempt success and need for cervical spine extension and use of Magill forceps.

	Video laryngoscopy *n* = 32	Direct laryngoscopy *n* = 32	OR	*p*-value
Rate of first trial success	31 (96.9%)	25 (78.1%)	8.68 [1, 75.3]	0.058
Number of 2^nd^ trial success	1 (3.1%)	7 (21.9%)		

Number of success rate	32 (100%)	32 (100%)	1	1

Need for use of Magill forceps	15 (46.9%)	29 (90.6%)	10.96 [2.77, 43.4]	0.0004

Need for cervical spine extension	9 (28.1%)	26 (81.2%)	11.07 [3.42, 35.88]	0.0005

Need for fibro optic	0 (0%)	0 (0%)	1	1

*Note:p* value was determined by chi square to compare the differences in frequencies between groups. Statistically significant: *p* value < 0.05.

Abbreviation: OR, odd′s ratio.

## Data Availability

The data that support the findings of this study are available on request from the corresponding author. The data are not publicly available due to privacy or ethical restrictions.
